# A Novel Karyoplasmic Ratio-Based Automatic Recognition Method for Identifying Glioma Circulating Tumor Cells

**DOI:** 10.3389/fonc.2022.893769

**Published:** 2022-05-13

**Authors:** Xinyi Zhu, Shen Wen, Shuhang Deng, Gao Wu, Ruyong Tian, Ping Hu, Liguo Ye, Qian Sun, Yang Xu, Gang Deng, Dong Zhang, Shuang Yang, Yangzhi Qi, Qianxue Chen

**Affiliations:** ^1^ Department of Neurosurgery, Renmin Hospital of Wuhan University, Wuhan, China; ^2^ School of Physics and Technology, Wuhan University, Wuhan, China; ^3^ Department of Circulating Tumor Cells, YZY (Youzhiyou) Medical Technological Company, Wuhan, China; ^4^ Department of Reagent Antibody, Genscript Biotech Corporation, Nanjing, China; ^5^ School of Electronic Information and Automation, Guilin University of Aerospace technology, Guilin, China; ^6^ Frontier Science Center for Immunology and Metabolism, Wuhan University, Wuhan, China

**Keywords:** circulating tumor cells, glioma, karyoplasmic ratio, clinical image, automatic recognition algorithm

## Abstract

**Background:**

Detection of circulating tumor cells (CTCs) is a promising technology in tumor management; however, the slow development of CTC identification methods hinders their clinical utility. Moreover, CTC detection is currently challenging owing to major issues such as isolation and correct identification. To improve the identification efficiency of glioma CTCs, we developed a karyoplasmic ratio (KR)-based identification method and constructed an automatic recognition algorithm. We also intended to determine the correlation between high-KR CTC and patients’ clinical characteristics.

**Methods:**

CTCs were isolated from the peripheral blood samples of 68 glioma patients and analyzed using DNA-seq and immunofluorescence staining. Subsequently, the clinical information of both glioma patients and matched individuals was collected for analyses. ROC curve was performed to evaluate the efficiency of the KR-based identification method. Finally, CTC images were captured and used for developing a CTC recognition algorithm.

**Results:**

KR was a better parameter than cell size for identifying glioma CTCs. We demonstrated that low CTC counts were independently associated with isocitrate dehydrogenase (IDH) mutations (*p* = 0.024) and 1p19q co-deletion status (*p* = 0.05), highlighting its utility in predicting oligodendroglioma (area under the curve = 0.770). The accuracy, sensitivity, and specificity of our algorithm were 93.4%, 81.0%, and 97.4%, respectively, whereas the precision and F1 score were 90.9% and 85.7%, respectively.

**Conclusion:**

Our findings remarkably increased the efficiency of detecting glioma CTCs and revealed a correlation between CTC counts and patients’ clinical characteristics. This will allow researchers to further investigate the clinical utility of CTCs. Moreover, our automatic recognition algorithm can maintain high precision in the CTC identification process, shorten the time and cost, and significantly reduce the burden on clinicians.

## Introduction

Circulating tumor cells (CTCs) are an important component of liquid biopsies and play an increasingly important role in cancer management (1–6). They have the advantage of non-invasive and convenient sampling and are commonly used for cancer diagnosis, dynamic monitoring of disease progression, and prognostic prediction. Undoubtedly, this technological advancement holds promise for further application in glioma, the deadliest brain tumor for which there is no safe monitoring method routinely. In particular, since scientists have successfully captured CTCs from the peripheral blood of patients with glioblastoma multiforme (GBM), World Health Organization (WHO) grade 4 glioma, a more comprehensive and in-depth understanding of glioma CTC characteristics has been achieved (7–9). However, the low detection efficiency with the currently available methods is still the most important factor limiting the clinical utility of CTCs in glioma.

Currently, the challenges of CTC detection are mainly focused on the two major issues of isolation and identification; correct identification is even more difficult than isolation. Because complete separation of CTCs and blood cells can barely be achieved by “positive enrichment,” “negative depletion,” or physical isolation, researchers need to further screen out CTCs from thousands of isolated nucleated cells. Among the various identification approaches, immunofluorescence (IF) staining is currently the most widely used technique (7–14). However, the use of antibodies against one or more protein surface markers will render the detection fragile to changes in the expression level of the selected marker, while targeting several proteins using an antibody cocktail will increase the risk of false-positive results and high background levels because healthy cells might express one or more of the included markers (7, 9, 11, 14). Thus, we believe that the development of other identification parameters will be an important component of IF staining for CTC identification. To address this issue, we proposed an identification method of IF staining combined with cell morphological features in our previous study, in which cell size was used as an important index for determination, which remarkably increased the specificity compared to IF staining alone (14). By using this identification strategy, the threshold of CTCs in healthy donors and detection level in glioma patients was 3.0 and 5.5 ± 3.0 (median: 5.0 CTCs, range: 0–13) CTCs per blood sample. It was noteworthy that our detection rate was as high as 85.7 (36/42 glioma patients), which was the highest yet reported. Although this strategy remarkably reduced background levels in healthy donors and increased the CTC detection rate, it also led to misidentification of a proportion of CTCs that were small in size. In particular, CTCs below the normal cell size have been increasingly reported, especially in highly heterogeneous GBM (15). Hence, evaluation of the role of other parameters (other morphological features) in distinguishing a broad repertoire of glioma CTCs from leukocytes is urgently required.

In addition, the interpretation of IF images is highly dependent on visual identification and annotation under the microscope by specialized physicians, who need to accurately discern 1–10 CTCs from thousands of nucleated cells, making this endeavor challenging. This is discussed in three aspects: First, there may be subtle differences in labeling results among CTCs owing to the use of antibody cocktail, as well as batch effects, easily leading to the occurrence of false-positive results (7, 13, 14); second, because of the background interference after the enrichment of CTCs (normal cells may express one or more of the targeted markers), the identification process needs to be combined with more parameters (including KR, cell volume, and nucleus morphology), further increasing the difficulty of microscopic identification through the naked eye; third, because of limited identification with human vision, misinterpretation of the results may be possible during macroscopic identification of CTCs under the microscope. In view of the above three demands and the remarkable progress made by artificial intelligence (AI) in the field of medical image recognition (16–18), we believe that developing computer technology and image processing technology to realize automatic identification and annotation of CTCs in gliomas would be of great clinical value for further application of CTCs.

Herein, after a comprehensive assessment of the roles of several morphological features in CTC identification in a previous study (19–21), we developed and validated a novel identification method to quantify CTCs isolated from peripheral blood samples of patients based on a method combining IF staining and KR, an important feature of malignant cells. Subsequently, we aimed to investigate the correlation between high-KR CTCs and patients’ clinical characteristics to evaluate clinical utility. Finally, based on acquired glioma CTC images and the aforementioned identifying parameters, we combined computer technology with digital image processing technology to construct an algorithm that mimics the CTC identification process by specialized physicians and achieve automated identification and annotation of CTCs in gliomas.

## Methods

### Blood Samples of Patients and Healthy Donors

Sixty-eight patients who were diagnosed with glioma underwent surgery and were enrolled in this study. After receiving written informed consent, peripheral blood samples were collected from patients and healthy donors under the Institutional Review Board-approved protocols. All patients in the study were free of significant comorbid medical conditions or prior cancer, deemed operable, and underwent a biopsy, subtotal, or gross total surgical resection (**Table 1**). Peripheral blood samples (5 ml × 2) were collected in EDTA buffer and processed by our ISET device (14) through the automatic isolation and staining procedure. All of the samples were collected before initial treatment and handled within 4 h. For postoperative patients, peripheral blood samples (5 ml × 2) were collected 2 weeks after surgery.

**Table 1:** Baseline information of patients.

Patients’ information	No. of cases (*n* = 68)
**Age (years)**	
>48	39
≤48	29
Sex	
Male	44
Female	24
**WHO grade**	
1	5
2	17
3	10
4	36
**Pathology**	
Astrocytoma	13
Oligodendrogliomas	13
GBM	33
Others	9
**IDH status**	
Mutant	21
Wild type	36
NA	11
**1p19q status**	
Co-deletion	13
Non co-deletion	44
NA	11
**CTC collection time**	
Before surgery	53
After surgery	28

NA, not available.

### Cell Lines

To establish the stable cell lines of U87-GFP and U251-GFP, a lentiviral plasmid, pLVX-GFP-puro (Miaolingbio, Wuhan, China), was transfected with two helper vectors, pMD2.G (#12259, Addgene, USA) and psPAX2 (#12260, Addgene, USA), into 293T cells using the Lipofectamine 2000 transfection reagent (Invitrogen, Carlsbad, CA, USA) to produce lentiviruses. The cells were infected with the lentiviruses and selected by puromycin (#73342, Stemcell, Canada) (5 mg/ml for U87 and 8 mg/ml for U251). The efficiency of viral infection was monitored by an Olympus BX51 microscope (Olympus, Tokyo, Japan).

### Isolation Procedure of the ISET Device

The blood sample (5 ml) was diluted 1:2 with BD wash buffer (BD, USA) containing 0.2% paraformaldehyde (PFA), 0.1% bovine serum albumin (BSA), and 0.0372% EDTA. It was incubated for 10 min at room temperature and then detected by our ISET device. Our ISET device and the protocols of isolation were introduced in detail in our previous study (14). Subsequently, the filtrate was gently aspirated by a vacuum suction pump. After aspiration, the retained cells were washed three times with pure water and fixed in 100% methanol. After disassembly from the filter, the membrane was placed on a slide and coverslipped after it had air-dried. The protocols of CTC isolation and detection were performed in our previous study (14).

### STEAM Staining

STEAM staining was a specific antibody cocktail against GBM CTCs developed by researchers in 2014 (7). Given the heterogeneity of GBM and the unknown expression profile of putative GBM CTCs, researchers sought to develop a cocktail of antibodies that would identify a broad spectrum of GBM cells, by searching the GBM biomarker literature and utilized publicly available microarray data on GBM tumors, cell lines, and purified WBC populations to identify GBM-specific markers. From this process, five antibodies were selected, based on their strong immunofluorescent staining of GBM cells and their complete absence in normal blood cells. This antibody cocktail, annotated as STEAM (SOX2, Tubulin beta-3, EGFR, A2B5, and c-MET), was combined into a single IF staining channel.

We fixed the captured cells on the membrane with 4% PFA for 5 min and subsequently washed it with PBS 3 times. Then, 150 μl of 0.3% Triton-X 100 was added for 3 min in order to allow for intracellular staining. After that, we added 10% goat serum (Jackson ImmunoResearch) to block nonspecific binding for half an hour. Then, we discarded the serum and added the primary rabbit antibodies against Sox2 (CST), EGFR (CST), Met (CST), A2B5 (Abcam), and Tubulin (Abcam); the mouse antibodies against CD14 (BD) and CD16 (Santa Cruz Biotechnology); and the rat antibody against CD45 (Santa Cruz Biotechnology) diluted 1:100 for incubation overnight at 4°C. On the next day, we washed the membrane with PBS 3 times and added the secondary Alexa Fluor 488 Goat anti-Rabbit IgG (Thermo Fisher), Alexa Fluor 546 Goat anti-Mouse IgG (Invitrogen), and Alexa Fluor 546 Goat anti-Rat IgG (Invitrogen) diluted 1:200 for incubation for 45 min at 37°C. The nuclei were stained with DAPI. The slides were imaged by an automated fluorescence microscopy scanning system (OLYMPUS IX81) under ×40 magnification.

### Low-Pass Whole-Genome Sequencing of CTC

Because the permeabilization process for STEAM staining is not compatible for the isolation of intranuclear DNA, CTCs were identified using fluorescently labeled antibodies against the surface markers: EGFR, MET, and CDH11. Subsequently, IF staining-positive CTCs were isolated by laser capture microdissection (LCM) technology. The minute amounts of DNA from CTC were amplified by the multiple annealing and looping-based amplification cycles (MALBAC) technique. DNA sequencing was performed by Illumina HiSeq 2500 system.

### Automatically Segmentation and Recognition Algorithm

Our CTC recognition algorithm is designed to imitate the process of manually counting CTC (**Figure 4**). First, the image is read and converted to a grayscale image (the 24-bit color JPEG image with a resolution of 1,920 × 1,440 is converted to an 8-bit grayscale image by reading only the grayscale values of the corresponding dye channel). Then, the nucleus is located in the blue channel (DAPI). The green channel (STEM) and the red channel (CD45) are segmented according to the location of the nucleus (**Figures 5A–C**). The KR and the proportion of the red part are calculated. The specific flow of our algorithm is as follows:

1. The image obtained by the CTC detection device is used as the input data of the algorithm, in which DAPI is input as the blue channel image, STEM is input as the green channel image, and CD45 is input as the red channel image, and is converted into a grayscale image.
2. Since the background levels in blue channel (DAPI) are relatively low, the Otsu method is directly used to divide each pixel of the blue channel into two categories: target and background, and the blue channel is binarized.
3. Find the connection region of the binarized image of the blue channel (DAPI) and obtain the maximum width (*W*) and maximum height (*H*) of each connected region.
4. Set the upper and lower thresholds of height (*h*1 and *h*2) and the upper and lower thresholds of width *w*1 and *w*2 and make conditional judgments: if *h*1 < *H* < *h*2 and *w*1 < *W* < *w*2, go to the next step. If there is no connected region that meets the above conditions, it is determined that there are no CTCs, and the picture is output (where *h*1 and *w*1 take the value slightly smaller than the diameter of the filter hole, and *h*2 and *w*2 take the value slightly larger than the distance between the two filter holes. In our picture, the filter hole diameter is about 56 pixels, *h*1 = *w*1 =46. The distance between the centers of two filter hole is 180 pixels, *h*2 = *w*2 = 200, which is used to remove large-scale pollution and reduce the amount of calculation).
5. Taking the width and height obtained in step (3) as parameters, enlarge the width and height by 3 times, respectively, and take the bounding box where the nucleus is located and mark it. We can get bounding boxes of varying numbers according to the number of nuclei.
6. Region growth in the bounding box is marked in the blue channel. Select the central 11 × 11 area of the bounding box and select the point where the grayscale value in this area is between 80% and 95% of the grayscale histogram of the entire bounding box as the seed point and begin growing based on the seed points. Choose the grayscale value with the most occurrences in the bounding box as the background grayscale in the bounding box. When a point in the 8 neighborhoods of the seed point matches the condition, (1) the difference between the grayscale value of the point and the grayscale value of the center point is less than the grayscale value of the center point multiplied by *p*; and (2) the grayscale value of the center point is greater than the background grayscale value plus the standard deviation of the grayscale value of the bounding box. Label the point as a target point and a new seed. Repeat the above process until no new seed point is added, and the blue channel growth ends. To enhance the connectivity of the image, morphological operations are implemented on the image, such as open and close operations to remove discrete points and fill holes.
7. According to the bounding box in the blue channel, the detection areas in the green channel and the red channel are locked, respectively, and regional growth is implemented within the bounding box. When generating the bounding box, it has been known that there must be a target in the bounding box of the blue channel, but there may not be a target in the bounding box of the green channel and the red channel. We need to find whether there is a target in the green channel and the red channel. First, select the grayscale value with the most occurrences in the whole picture as the global background grayscale value, and then choose the grayscale value with the most occurrences in the bounding box as the local background grayscale value in the bounding box. Calculate the average grayscale value of the target in the same position in the green channel and red channel in the binarized blue channel generated in step (6). When the bounding box of the green channel and the red channel matches the condition, (1) the difference between the average grayscale value and the global background grayscale value is less than the standard deviation of the global grayscale value; and (2) the difference between the average grayscale value and the local background grayscale value is less than the standard deviation of the bounding box. It is considered that there is no target in this channel. If there is a target in the channel, the same region growth as in step (6) is performed. Because it has been obtained, the binarized blue channel in step (6), the area for selecting the seed point, needs to change from an 11 × 11 area in the center of the bounding box to the target area of the blue channel, which is generated in step (6).
8. Calculate karyoplasmic ratio (*KR*) and the proportion of red (*Red_prop*) according to the binarized DAPI, STEAM, and CD45 images segmented in steps (6) and (7).
9. Using the result of step (7) to compare with the threshold, the threshold of the KR and the proportion of red are set to *K* and *R*, respectively. Cells with a proportion of red higher than *R* were considered to have a common leukocyte antigen, and cells with KR less than *K* were considered non-CTCs. The output bounding box binarized image and calculation results. After processing all bounding boxes in the picture, the CTCs were marked with a red box and the picture was output.

### Evaluation Criteria for Our Classification Algorithm

After the segmentation of these images, some performance evaluation criteria were involved to evaluate the performance of our classification algorithm. There are four basic categories—True Positives (TP), False Positives (FP), True Negatives (TN), and False Negatives (FN)—that are commonly used to describe the overlap of predictions with ground-truth labels. True means the prediction is correct, False means the prediction is wrong, Positive means the label is CTC, and Negative means the label is non-CTC. Here, we choose five metrics—accuracy, sensitivity (Se or recall), specificity (Sp), precision, and F1 score—to evaluate the performance of our classification algorithm.


$$Accuracy=\frac{TP+TN}{TP+TN+FP+FN}$$

$$Sp=\frac{TN}{TN+FP}$$

$$Se\left(recall\right)=\frac{TP}{TP+FN}$$

$$Precision=\frac{TP}{TP+FP}$$

$$\text{F}1=\frac{2\times Precision\times Se}{Precision+Se}=\frac{2\times TP}{2TP+FP+FN}$$

### Statistical Analysis

Statistical analysis was mainly performed with R (https://www.r-project.org/) with several publicly available packages. *p* < 0.05 was considered to indicate statistical significance (*p < 0.05, **p < 0.01, ***p < 0.001, and ****p < 0.0001, as indicated in the figures and legends).

## Results

### Confirmation of Identification of Cells of Smaller Size Through Copy Number Variation detection

First, of the 10 patients with gliomas of WHO grades 2–4 enrolled in this study, we focused on whether IF staining-positive cells with a small size (<16 μm) belong to CTCs. In particular, we found that “smaller CTCs” were more often observed in GBM, a WHO grade 4 glioma (**Figure S1**).

**Figure 1:**
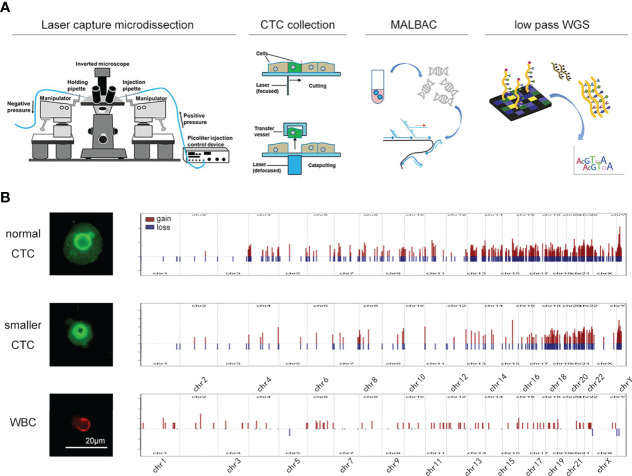
The isolation and sequencing of glioma CTC. **(A)** Schematic of the experimental design. CTCs were collected by LCM technology and amplification was performed by MALBAC technology. The amplified products were used to perform DNA-seq. **(B)** The CNV pattern in normal CTC (upper), smaller CTC (middle), and leukocytes (lower).

To clarify this subtype of CTCs, we utilized a single-cell DNA sequencing technique to analyze its variation patterns (**Figure 1A**). Because the permeabilization process for STEAM staining is not compatible with the isolation of intranuclear DNA, CTCs were identified using fluorescently labeled antibodies against the surface markers EGFR, MET, and CDH11 (7). Single-cell low-pass whole-genome sequencing (lp-WGS) analysis revealed that copy number variation in “smaller CTCs” was highly similar to that in normal CTCs, whereas it was quite different from that in leukocytes (**Figure 1B**). This result was consistent with that of a previous study and urged us to focus on smaller CTCs. Diffuse glioma is a highly heterogeneous disease with different tumor cell sizes. In particular, its heterogeneity might increase with an increase in tumor malignancy, leading to various sizes of CTC in high-grade gliomas. Our findings are consistent with this phenomenon. Therefore, other morphological features of malignant cells must be evaluated to develop a new CTC identification standard.

### Defining Karyoplasmic Ratio as an Important Parameter in CTC Identification

**Figure 2:**
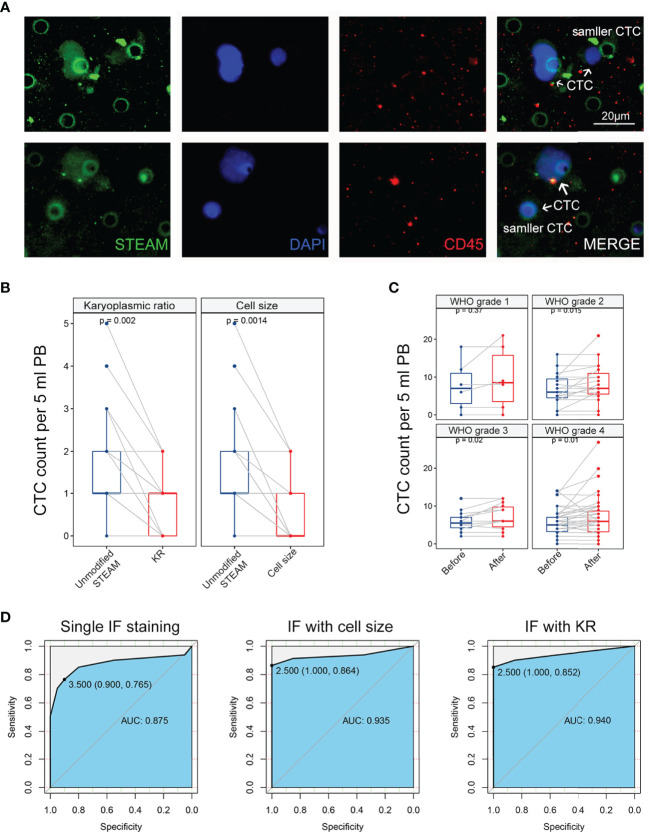
Comparing identification effect of methods based on karyoplasmic ratio and cell size in clinical samples. **(A)** The representative IF image of CTC in glioma, containing CTC with normal size and smaller size. Scale bar = 20 μm. **(B)** Left panel: method based on karyoplasmic ratio decreased the background level and false-positive risk in healthy donors (*p* = 0.002). Right panel: method based on cell size also decreased the background level and false-positive risk in healthy donors (*p* = 0.0014). **(C)** Compared with the method based on cell size (blue), the method based on karyoplasmic ratio significantly increased detectable level of CTC in glioma with WHO grade 2-4 (*p* = 0.015, *p* = 0.02, and *p* = 0.01, respectively). Before: method with cell size; after: method with KR. **(D)** Left panel: ROC curve for single IF staining in glioma diagnosis (AUC = 0.875). Middle panel: ROC curve for IF staining with cell size in glioma diagnosis (AUC = 0.940). Right panel: ROC curve for IF staining with KR in glioma diagnosis (AUC = 0.935).

Notably, after DAPI staining and antibody labeling, larger nuclei were observed in STEAM^+^/CD45^-^ cells than in STEAM^-^/CD45^+^ leukocytes (**Figure 2A**). Even in cells that were smaller in size, their nuclei appeared significantly larger than those in the cytoplasm (**Figure 2A**). Dysregulated KR is among the most representative feature of tumor cells owing to uncontrolled division that makes the nucleus grow much faster than the cell does. Therefore, we deduced that abnormal KR may play an important role in CTC identification.

To verify our conjecture, 20 blood samples collected from glioma patients (5 patients each in WHO grades 1–4) were analyzed for further details. Peripheral blood was collected using our ISET isolation device, and enriched cells were marked with an antibody cocktail annotated as STEAM and antibody against CD45 as well as DAPI. By converting the raw image to a grayscale image and calculating the area of DAPI and STEAM staining, a new parameter, the ratio of the area of the nucleus to that of the cytoplasm, was defined as KR of the cells. Using this new parameter, cells labeled with a single nucleus were divided into two categories. In the STEAM^+^/CD45^-^ cell group, the vast majority showed a high KR (HKR), while very few showed a low KR (LKR). Corresponding to this, the STEAM^-^/CD45^+^ group showed LKR. These results indicated that HKR cells were more likely to be tumor cells.

For the quantification of KR in CTCs and white blood cells, we developed an algorithm based on the calculation of the IF-labeled area (KR = DAPI area/total cell area). In the above samples, we found that the KR of STEAM^+^/CD45^-^ cells was 0.807 ± 0.055, 0.821 ± 0.065, 0.787 ± 0.047, and 0.878 ± 0.046, respectively, ranging from WHO grades 1 to 4, while the KR of STEAM^-^/CD45^+^ cells was 0.450 ± 0.031, 0.449 ± 0.082, 0.396 ± 0.061, and 0.531 ± 0.041, respectively. In blood samples from five healthy donors, the KR of STEAM^+^/CD45^-^ cells was 0.848 ± 0.039, whereas that of STEAM^-^/CD45^+^ cells was 0.425 ± 0.044. Furthermore, we selected two glioma cell lines for quantification. The KR of U87 cell lines was 0.802 ± 0.059 and that of U251 was 0.772 ± 0.042 (**Table 2**). In addition, the threshold of HKR cells was similar to that in Liu’s study (19), revealing that KR might be a much more reliable identification parameter for glioma CTCs.

**Table 2:** Quantization of karyoplasmic ratio in clinical samples and cell lines.

Group	STEAM^+^/CD45^-^		STEAM^-^/CD45^+^	
	Count	Karyoplasmic Ratio	Count	Karyoplasmic Ratio
WHO 1 grade	46	0.807 ± 0.055	1000	0.450 ± 0.031
WHO 2 grade	50	0.821 ± 0.065	1000	0.449 ± 0.082
WHO 3 grade	43	0.787 ± 0.047	1000	0.396 ± 0.061
WHO 4 grade	54	0.878 ± 0.046	1000	0.531 ± 0.041
Healthy donors	9	0.848 ± 0.039	1000	0.425 ± 0.044
U87 cell line	10,000	0.802 ± 0.059	0	–
U251 cell line	10,000	0.772 ± 0.042	0	–

Karyoplasmic ratio = DAPI area/total cell area; Data are presented as the mean ± SD of independent experiments.

### Comparing the Identification Effect of Methods Based on Karyoplasmic Ratio and Cell Size in Clinical Samples

First, the development of a KR-based identification method for CTCs was aimed at reducing the risk of false-positive results and high background levels. To further study this strategy, blood samples obtained from 20 healthy donors were randomly selected and tested using the same methods. In our previous study, we demonstrated that this identification strategy was able to reduce the background levels in healthy donors using a cell size identification method. Interestingly, when we used KR as an ancillary identification method, we observed that a subset of cells with LKR was present in STEAM^+^/CD45^-^ CTCs from the peripheral blood of healthy donors (**Figure 2A**). Using the KR-based identification strategy, the detectable number of STEAM^+^/CD45^-^/HKR cells was remarkably decreased in healthy donors (**Figure 2B**). In addition, no significant difference in the decrease in the background level was observed between the two identification methods (*p* > 0.05). Because the limitation of the antibody cocktail for several surface markers mainly focuses on the high background level and false-positive results (7, 11, 14), our findings indicate that this strategy could overcome the drawbacks of targeting several proteins and be an important complement of IF staining in CTC identification.

Second, our KR-based identification strategy has the advantage of increasing the detection rate by detecting CTCs of smaller sizes. Thus, we further validated it in clinical samples to compare the detection level of CTCs using the above two methods. Among 68 glioma patients, with an increase in tumor malignancy and WHO grade, the detectable level of CTCs was remarkably increased (**Figure 2C**). For WHO grade 2 glioma, the detectable level of CTCs increased from 6.7 (median: 6 cells, range: 0–16, mean: 6.7 ± 4.2) to 8.4 (median: 7 cells, range: 0–21, mean: 8.4 ± 5.1) (*p* = 0.015). For patients with WHO grade 3, the detectable level of CTCs increased from 5.9 (median: 5.5 cells, range: 2–12, mean: 5.9 ± 3.7) to 7.1 (median: 6 cells, range: 2–12, mean: 7.1 ± 3.4) (*p* = 0.02). For GBM, the detectable level of CTCs increased from 6.1 (median: 5.5 cells, range: 0–14, mean: 6.1 ± 3.3) to 7.4 (median: 6 cells, range: 0–27, mean: 7.4 ± 5.3) (*p* = 0.01). To further evaluate the efficiency of three identification methods in glioma diagnosis, we used an ROC curve to determine sensitivity and specificity. The ROC curve showed that the area under the curve (AUC) of single IF staining, IF with cell size, and IF with KR were 0.875, 0.940, and 0.935, respectively (**Figure 2D**). These results indicate that the KR-based method can increase the sensitivity of CTC identification compared to the cell size-based method. Therefore, further studies on the relationship between CTC counts and patients’ clinical characteristics are required to reevaluate their clinical utility.

### Correlation Between CTCs and Clinical Characteristics in Glioma Patients

When validated in clinical samples, we found that the detectable level of CTCs in the peripheral blood of glioma patients was not related to the WHO grade of the tumor, which was consistent with the finding of a previous study (10, 14). One explanation is that the high heterogeneity of high-grade gliomas results in the loss of our targeted markers, leading to CTC capture being a small proportion of total glioma CTCs. Another explanation is that the group of determinant CTCs had not been precisely identified, exemplified by the fact that only vimentin-positive CTCs were related to patients’ outcomes rather than EpCAM-positive CTCs in colon cancers (22). In addition to the aforementioned potential cases, the specificity of CTC detection in brain tumors using peripheral blood should also be considered. Although CTCs have been detected in the peripheral blood of glioma patients, their clinical significance remains unknown. On the one hand, GBM-related tumor metastases were rare; on the other hand, it remains debatable whether the detection of CTCs using 5 ml of peripheral blood was able to reflect the situation in 5 L of whole blood.

**Figure 3:**
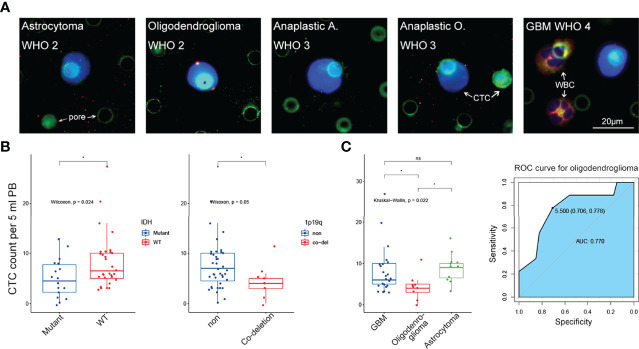
The correlation between CTC level and clinical characteristics. **(A)** Representative IF image of CTC in 5 subtypes of glioma from WHO grade 2 to grade 4. Scale bar = 20 μm. **(B)** Left panel: low CTC level was significantly related to IDH mutant status (*p* = 0.024). Right panel: low CTC level was significantly related to 1p19q co-deletion status (*p* = 0.05). **(C)** Left panel: CTC level in oligodenroglioma was significantly lower than that in astrocytoma and GBM (*p* < 0.05 and *p* < 0.05, respectively). Right panel: ROC curve revealed that low CTC level was a good predictor for oligodenroglioma pathological subtype (AUC = 0.770). ns, no significance.

However, we could still observe a close relationship between CTC counts and patients’ histopathology and molecular pathology (**Figures 3A, B**). Consistent with the findings of our previous research, low CTC counts were found in patients with an isocitrate dehydrogenase (IDH) mutant status (*p* = 0.024). We also observed that patients with 1p19q co-deletion status were more likely to have low CTC counts (*p* = 0.05). IDH and 1p19q status have important roles in the molecular diagnosis of diffused glioma (23, 24). Therefore, we further investigated the correlation between CTC counts and glioma histopathology. We found that oligodendroglioma (WHO grades 2–3, IDH mutant, and 1p19q co-deletion) showed a remarkably lower CTC count than astrocytoma (WHO grades 2–4, *p* = 0.011) and GBM (WHO grades 2–4, IDH wild type, *p* = 0.043) (**Figure 3C**). Despite the lack of correlation between CTC counts and tumor malignancy, we still demonstrated the potential application of CTCs in tumor diagnosis. These results highlight the potential value of CTCs in glioma classification, including histopathological and molecular pathological diagnosis.

### Development of an HKR-Based CTC Identification Assay Using an Automatic Segmentation and Recognition Algorithm

**Figure 4:**
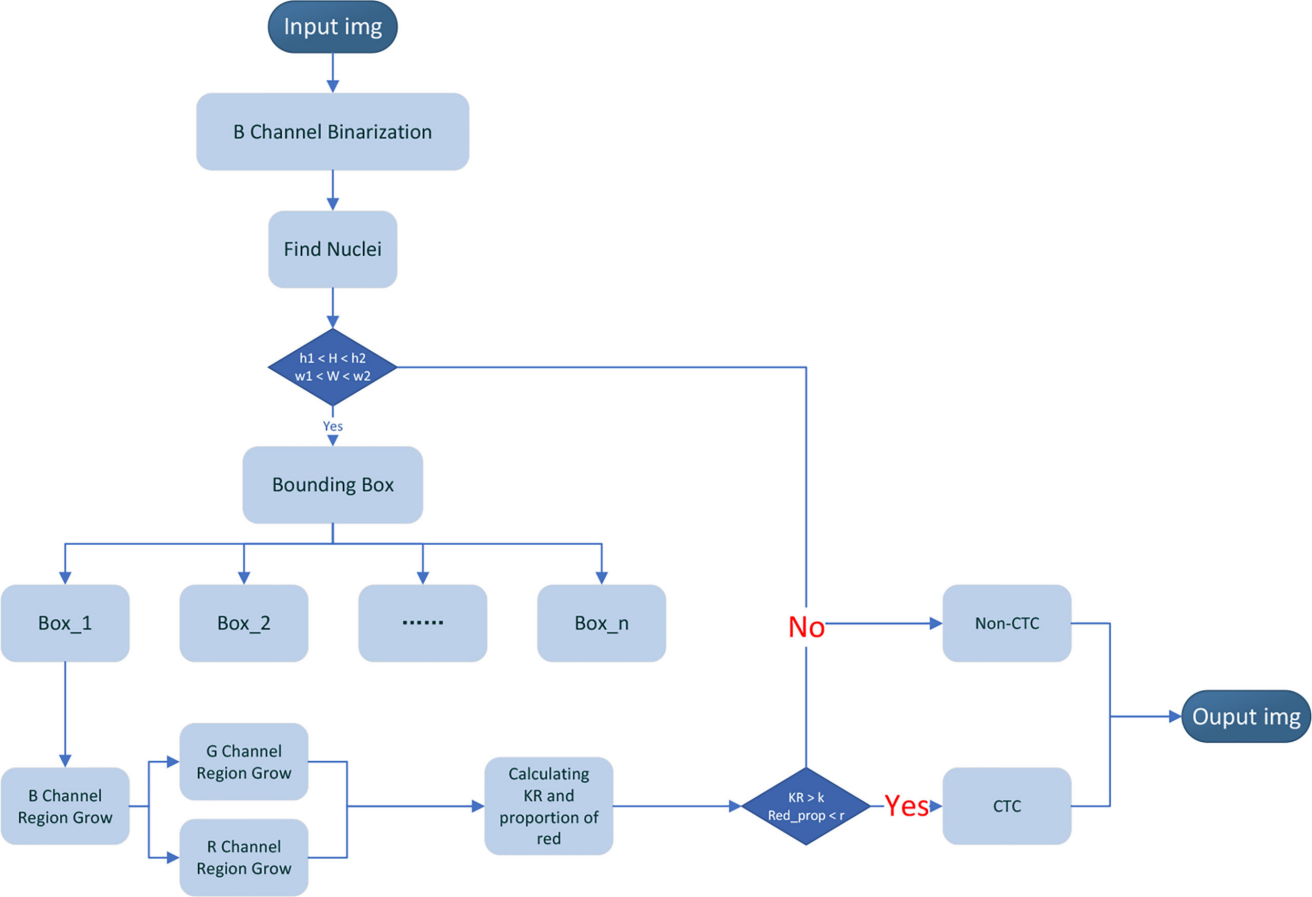
Flowchart of automatic CTC recognition algorithm. Schematic of the algorithm design.

**Figure 5:**
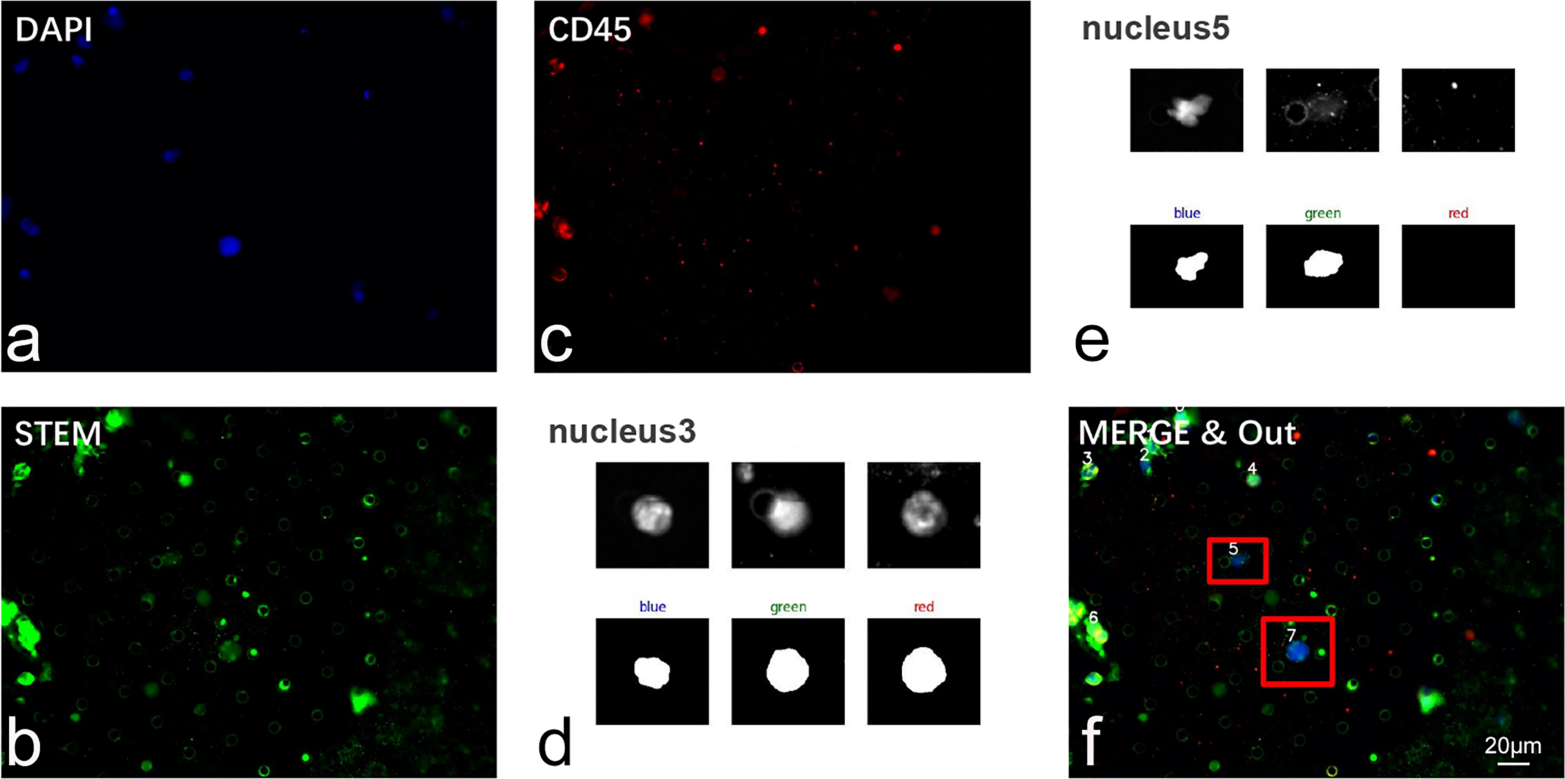
Validation of CTC recognition algorithm in clinical samples. **(A–C)** Representative IF image of cells isolated from glioma patients’ peripheral blood. Cells were sequentially labeled by DAPI (blue), STEAM (green), and CD45 (red). **(D, E)** Automatic segmentation of nucleated cells. **(F)** Automatic recognition of CTC through our algorithm. CTCs were marked by red anchor box. Scale bar = 20 μm.

For the precise localization of CTCs and the accurate calculation of the above-mentioned parameters, we developed an algorithm for automatic segmentation and identification based on CTC IF images (**Figure 4**). Because a full image is extremely large, the complexity of the algorithm is positively related to the size of the image. To reduce the number of calculations, we selected part of the image to perform the following tests. There may be only one CTC or multiple CTCs in these images and possibly other non-CTCs. The resolution of the selected part of the image was 1,920 × 1,440. Our CTC recognition algorithm was designed to imitate the manual counting of CTCs. First, the image is read and converted into a grayscale image. The nuclei were then located in a blue channel (DAPI). The green (STEAM) and red (CD45) channels were segmented according to the location of the nucleus (**Figures 5A–C**).

Together with the findings described above, we incorporated both the calculation of KR and the co-expression profile of CD45 into the CTC identification standard. A previous study reported that the nucleus with a proportion of red higher than 30% was commonly defined as having a common leukocyte antigen (25). In this study, because we used an automatic recognition algorithm, filter pore and impurities on the images may interfere with calculation of proportion of CD45 and thus affect the final identification results. To quantify the threshold of HKR and CD45 co-expression profiles, 337 images containing 353 CTCs and 1,271 leukocytes were labeled for calculation. After referring to previous studies and our clinical data (**Table 2**), we established a standard for CTC: cells with a CD45 proportion of less than 35% and KR of >70% were considered CTCs (20).

### Validation of Our CTC Recognition Algorithm Using Clinical Samples

To further validate the clinical utility of our CTC recognition algorithm, we applied it to 197 images with at least one CTC, out of which there were 258 CTCs and 809 non-CTCs. The results obtained using our segmentation and recognition algorithms are listed (**Table 3**). The accuracy, sensitivity, and specificity were 93.4%, 81.0%, and 97.4%, respectively, whereas the precision and F1 score reached 90.9% and 85.7%, respectively, indicating that our CTC recognition algorithm was able to achieve automated identification and annotation of CTC in gliomas. Compared with the study by He et al. (21), our algorithm showed higher accuracy and specificity, indicating that our CTC recognition algorithm might perform better than machine learning in the case of small sample sizes.

**Table 3:** Validation of CTC recognition algorithm in clinical samples.

	Positive	Negative	Total
True	209	788	997
False	21	49	70

## Discussion

The present study showed that KR was a better parameter for detecting glioma cells than cell size. The accuracy, sensitivity, and specificity of the developed algorithm were 93.4%, 81.0%, and 97.4%, respectively.

Although CTCs are a promising technology for the diagnosis and monitoring of glioma, they play a key role in improving patient outcomes, and the isolation and identification of CTC have been proven challenging (7–14). With advances in technology, the capture efficiency of CTCs has greatly improved. However, the slow development of CTC identification methods has not received sufficient attention, which might hinder their further application. Currently, the identification of glioma CTC mainly faces two issues: (1) it is difficult to identify CTCs with high efficiency by single IF staining due to the highly heterogeneous nature of GBM; and (2) the interpretation of IF images is highly dependent on visual identification and annotation under the microscope by specialized physicians, which can easily produce errors.

To improve the identification efficiency of glioma CTCs, we developed and validated a novel identification method to quantify CTCs isolated from peripheral blood samples based on a combination of IF staining and KR, an important morphological feature of tumor cells. We found that this identification method remarkably increased the detection level of CTCs, compared with that reported in previous studies (7–11, 14). Subsequently, we combined computer technology with digital image processing technology to construct an algorithm that mimics the process of CTC identification by specialized physicians and achieve the automated identification and annotation of glioma CTCs.

This study is significant on four major fronts. First, this study identified a potential reason for the low efficiency of detecting glioma CTCs. Although CTCs are generally considered larger than 16 μm, CTCs below the normal size have been increasingly reported in several solid tumors (15). Our study confirmed the same phenomenon in glioma CTCs, particularly in GBM. These findings will contribute to a more efficient CTC detection strategy for evaluating the clinical utility of glioma CTCs.

Second, we developed a novel KR-based IF staining strategy for glioma CTC identification. Dysregulated KR is among the most representative features of tumor cells, owing to uncontrolled division, which makes the nucleus grow much faster than the cell does (19–21). A previous study proposed a KR-based imaging flow cytometry for the detection of CTCs in hepatocellular carcinoma and pointed out that the technique relying on the KR had a higher sensitivity than traditional techniques relying on antibodies or cell surface markers (20). However, this study relied on imaging flow cytometry, which was not a first-line method for isolating CTCs and might have led to biased results. Therefore, we combined IF staining and the KR-identifying technique in subsequent studies. We demonstrated that our KR-based IF staining strategy could significantly increase detection efficiency in glioma patients while reducing background levels in healthy donors. This finding may open a new window for glioma CTC detection and application, as only a sufficient number of CTCs can shed light on its clinical value.

Third, this study further revealed a correlation between CTC counts and patients’ clinical characteristics. Unlike previous studies (10), we confirmed the potential relationship between CTCs and tumor pathological diagnosis, including histopathology and molecular pathology. Based on our previous study (14), we further demonstrated the correlation between CTC counts, IDH status, and 1p19q status of glioma. IDH and 1p19q are two of the most important molecular pathological markers of glioma, which are closely related to tumor classification and patient outcomes (23, 24). We observed a remarkably lower CTC count in glioma patients with IDH mutation and 1p19q co-deletion status, which are typical features of oligodendrogliomas, suggesting that CTC count might be a good predictor for histopathological and molecular pathological diagnosis of glioma. Moreover, the IDH status plays an important role in predicting patient prognosis. However, limited by the short follow-up period, this study did not reveal a correlation between CTC counts and patient survival. Future work should further expand the sample size and refine the follow-up of patients to further evaluate the clinical utility of CTCs in glioma. It is also worth noting that the correlation between CTC count and tumor malignancy (from WHO grades 2 to 4) has not yet been observed, contrary to the favorable relationship between CTC counts and tumor molecular pathology. This may be because the group of determinant CTCs has not been precisely identified, as exemplified by the fact that only vimentin-positive CTCs were related to patient outcomes rather than EpCAM-positive CTCs in colon cancers (22). This finding urges investigators to further explore the subtypes of glioma CTCs that are decisive in predicting patient survival in future studies. In addition to the aforementioned potential reasons, the specificity of CTC detection in brain tumors using peripheral blood should also be considered. Although CTCs have been detected in the peripheral blood of glioma patients, their clinical significance remains unknown. On the one hand, GBM-related tumor metastases were rare; on the other hand, it remains debatable whether the detection of CTCs in 5 ml of peripheral blood could reflect the situation in 5 L of whole blood. Recent research has pointed out that cerebrospinal fluid (CSF) is a more appropriate sample for the liquid biopsy of brain tumors (26–29). Therefore, further detection of CTCs in CSF may have a positive effect in clarifying their clinical significance.

Finally, this study established an algorithm for automatic segmentation and recognition of glioma CTCs. Undoubtedly, machine-learning-based AI has made remarkable progress in the field of image recognition (16–18). To date, there have been some deep-learning-based methods for CTC recognition. However, these methods are based on complex neural networks, which require high-performance computers to accelerate computation and large amounts of data so that the networks have better generalization performance. Despite the extremely poor prognosis of glioma, its incidence remains relatively lower than that of breast and lung cancers, making it difficult to obtain a large amount of data for machine learning. By contrast, our CTC recognition algorithm requires much less computation, only requires tuning a few parameters, and does not require a large amount of labeled data for training. This automatic recognition algorithm can maintain high precision in the CTC identification process, shorten the time and cost, and greatly reduce the burden on clinicians.

There were still some limitations in this study. First, although our recognition algorithm showed high efficiency in small samples (<600 images) compared with AI, machine-learning-based AI remained the better method for identifying CTC images when the sample size is large enough. Second, limited by the short follow-up period, this study did not reveal a correlation between CTC counts and patient survival. Third, the correlation between CTC level and clinical characteristics remained unknown. Therefore, future work should focus on three aspects: (1) Continued expansion of clinical samples and research cohorts with close follow-up should be performed to investigate the relationship between CTCs, clinical characteristics, and patients’ survival. (2) Deep-learning-based methods are still a more accurate approach for the recognition of medical images than our simple algorithm because AI can obtain a larger amount of information that human eyes cannot capture from images. Therefore, future work is still needed to rationally utilize AI technology after obtaining sufficient CTC images to achieve the precise identification of CTCs and precision medicine for patients. (3) Last but not least, targeting CSF-derived CTCs might help to better understand the role of CTCs in glioma malignant behavior.

## Conclusion

In summary, we demonstrated that smaller CTCs (<16 μm) accounted for a proportion of glioma CTCs and were an important factor contributing to the low efficiency of the previous assays. We developed a novel KR-based IF staining strategy for glioma CTC identification, which remarkably increased the detection rate and reduced the number of false-positive events. Our findings also revealed a correlation between CTC counts and molecular pathological diagnosis of glioma. We established an algorithm for automatic segmentation and recognition of glioma CTCs. Based on our research, the KR-based automatic recognition method could simplify the identification process, increase the detection efficiency, and provide a bridge for further clinical application of CTCs in tumor management. Future work shall focus on utilizing AI to precisely identify images and investigate the correlation between CTC and patients’ clinical characteristics in larger clinical samples.

## Data Availability Statement

The datasets presented in this study can be found in online repositories. The names of the repository/repositories and accession number(s) can be found at: NCBI, SRA 11273088.

## Ethics Statement

The studies involving human participants were reviewed and approved by Institutional Review Board-approved protocols of the Renmin Hospital of Wuhan University. Written informed consent to participate in this study was provided by the participants’ legal guardian/next of kin. Written informed consent was obtained from the individual(s), and minor(s)’ legal guardian/next of kin, for the publication of any potentially identifiable images or data included in this article.

## Author Contributions

SY, YQ, and QC designed this study. XZ, SD, GW, QS, and YQ performed the isolation and identification of CTC. LY, GW, RT, and YQ performed MALBAC amplification, lp-WGS, and bioinformatics analysis. SW, SY, and DZ wrote the automatic segmentation and recognition algorithm. SD, GD, YX, and QS performed the clinical data collection and collation. All the authors were involved in the analysis and interpretation of data. XZ and YQ wrote the paper, with the help of the co-authors. SY and QC reviewed and revised the manuscript. All authors contributed to the article and approved the submitted version.

## Funding

The present study was supported by the National Natural Science Foundation of China (no. 82072764) (to QC) and the Fundamental Research Funds for the Central Universities (no. 2042022kf1106) (to YQ).

## Conflict of Interest

Author GW was employed by YZY Medical Technological Company. Author RT was employed by Genscript Biotech Corporation.

The remaining authors declare that the research was conducted in the absence of any commercial or financial relationships that could be construed as a potential conflict of interest.

## Publisher’s Note

All claims expressed in this article are solely those of the authors and do not necessarily represent those of their affiliated organizations, or those of the publisher, the editors and the reviewers. Any product that may be evaluated in this article, or claim that may be made by its manufacturer, is not guaranteed or endorsed by the publisher.

## Supplementary Material

Supplementary Figure 1 Detection of smaller CTC in patients with glioma. No significant difference was observed in detection level of smaller CTC in patients from WHO grade 1 to 4 (*p* > 0.05).
