# Supplementary figures and images for: A Novel Karyoplasmic Ratio-Based Automatic Recognition Method for Identifying Glioma Circulating Tumor Cells
Source: Front Oncol. 2022 May 13;12:893769. doi: 10.3389/fonc.2022.893769 (PMC9137408; doi:10.3389/fonc.2022.893769)

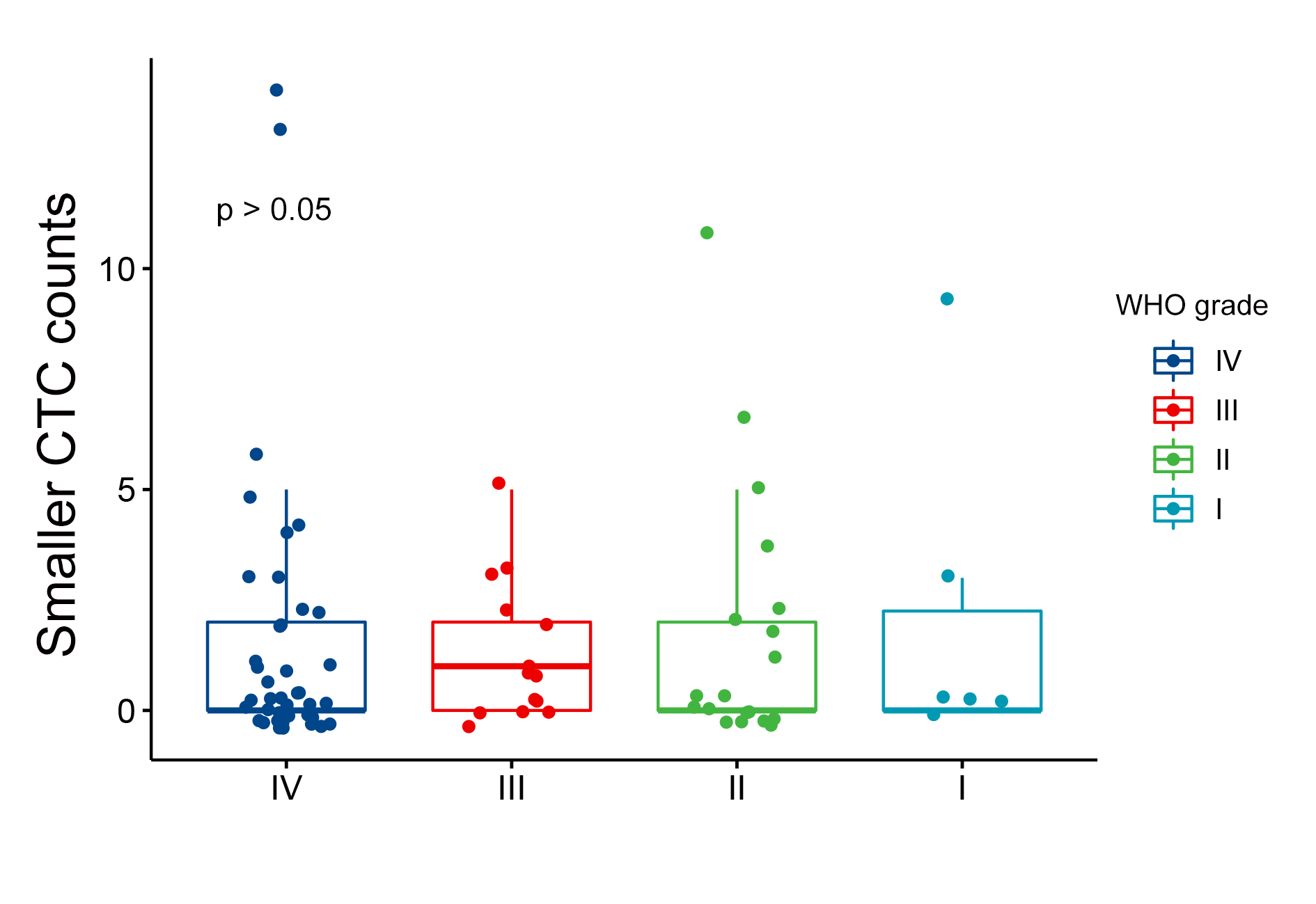

Supplement: Supplementary Figure 1 — Detection of smaller CTC in patients with glioma. No significant difference was observed in detection level of smaller CTC in patients from WHO grade 1 to 4 (p > 0.05). [file Image_1.tif]
